# Postoperative Changes in Fecal Bacterial Communities and Fermentation Products in Obese Patients Undergoing Bilio-Intestinal Bypass

**DOI:** 10.3389/fmicb.2016.00200

**Published:** 2016-02-23

**Authors:** Vania Patrone, Elia Vajana, Andrea Minuti, Maria L. Callegari, Alessandro Federico, Carmela Loguercio, Marcello Dallio, Salvatore Tolone, Ludovico Docimo, Lorenzo Morelli

**Affiliations:** ^1^Instituto di Microbiologia, Facoltà di Scienze Agrarie, Alimentari e Ambientali, Università Cattolica del Sacro CuorePiacenza, Italy; ^2^Istituto di Zootecnica, Facoltà di Scienze Agrarie, Alimentari e Ambientali, Università Cattolica del Sacro CuorePiacenza, Italy; ^3^Division of Hepatogastroenterology, Second University of NaplesNaples, Italy; ^4^Division of General and Bariatric Surgery, Second University of NaplesNaples, Italy

**Keywords:** obesity, gut microbiota, weight loss, bariatric surgery, short chain fatty acids, illumina sequencing, 16S rRNA

## Abstract

We assessed the gut microbial ecology of 11 severely obese patients before and after bilio-intestinal bypass (BIB). Fecal samples were evaluated for microbial communities using 16S rDNA Illumina sequencing, real-time PCR targeting functional genes, and gas chromatography of short chain fatty acids (SCFAs). At 6 months after surgery, subjects exhibited significant improvements in metabolic markers (body weight, glucose, and lipid metabolism) compared with baseline. The fecal microbiota of post-surgery individuals was characterized by an overall decrease of bacterial diversity, with a significant reduction in Lachnospiraceae, Clostridiaceae, Ruminococcaceae, Eubacteriaceae, and Coriobacteriaceae. On the contrary, there were significant increases of genera *Lactobacillus, Megasphaera*, and *Acidaminococcus* and the family Enterobacteriaceae. The pH was decreased in fecal samples from patients after BIB and SCFA profiles were altered, with lower percentages of acetate and propionate and higher levels of valerate and hexanoate. Some changes in the bacterial populations were associated with variations in the patients' metabolic health parameters, namely *Gemmiger* and glucose, *Lactobacillus* and glucose, and *Faecalibacterium* and triglycerides. The results from this study of BIB patients furthers our understanding of the composition of gut microbiota and the functional changes that may be involved in improving obesity-related conditions following weight-loss surgery.

## Introduction

Emerging evidence suggests that microbes that reside in the human gastrointestinal tract may be involved in the development of obesity. The gut microbiota contributes to weight gain by promoting storage of calories from the diet as fat (Bäckhed et al., 2004; Turnbaugh et al., 2006). Several studies in humans and animal models have highlighted differences in the composition of the gut microbiota between obese and lean subjects, sometimes yielding contrasting results (Ley et al., 2005, 2006; Turnbaugh et al., 2006, 2008, 2009; Schwiertz et al., 2010).

In addition, the association between gut microbiota and obesity is supported by studies exploring the effects of bariatric surgery on gut microbial community composition. This surgery is the most effective current option for treating severe obesity. By reducing intake or absorption of calories, surgical intervention produces rapid and long-lasting weight loss and leads to an improvement of many obesity-related conditions including type-2 diabetes, hypertension, and high cholesterol. Zhang et al. (2009) showed that individuals subjected to Roux-en-Y gastric bypass (RYGB), the most commonly performed bariatric procedure, have higher numbers of Enterobacteriaceae bacteria (phylum Proteobacteria) and lower proportions of *Clostridium* bacteria (phylum Firmicutes) and methanogens compared with obese individuals. A subsequent study by Furet et al. (2010) included obese subjects before, and 3 and 6 months after RYGB, along with lean controls. Their results revealed that the prevalence of the *Bacteroides*/*Prevotella* group was lower in obese individuals compared with lean subjects, but it increased after gastric surgery. On the contrary, populations of *Bifidobacterium* and *Lactobacillus*/*Leuconostoc*/*Pediococcus* decreased after surgery. At the phylum level, obese patients had a high Firmicutes to Bacteroidetes ratio before RYGB that decreased 3 and 6 months postoperatively.

Li et al. (2011) evaluated the effects of RYGB in a non-obese rat model, and found a striking increase of Proteobacteria as well as a decrease of both Firmicutes and Bacteroidetes compared with sham-operated rats. In a key study, Liou et al. (2013) performed 16S rDNA sequencing in three groups of mice, which underwent RYGB, sham surgery, or sham surgery coupled with caloric restriction. The RYGB produced specific changes in the gut microbial community, i.e., increases in Bacteroidetes, Verrucomicrobia, and Proteobacteria (*Alistipes, Akkermansia*, and *Escherichia*, respectively, at the genus level) compared with preoperative levels. Even more important, transferring gut microbiota from RYGB-mice to germ-free mice induced weight loss in those animals compared with mice receiving gut samples from sham-treated mice. These results suggest that changes in the population of microbial organisms in the gastrointestinal tract may mediate some of the beneficial effects of bariatric surgery on the obese host.

Although RYGB is the most widely used type of bariatric surgery in the world, there are several available surgical methods for the treatment of morbidly obese patients. Bilio-intestinal bypass (BIB) surgery (Eriksson, 1981) consists of disabling a large portion of the absorptive surfaces within the small bowel using a shunt between the beginning and end-part, and connecting the disabled small bowel sling to the gall-bladder to improve enterohepatic circulation of bile acids. It is unknown if the changes in microbial ecology observed for RYGB can be generalized to other types of bariatric surgery; therefore, we studied fecal samples using 16S rDNA next generation sequencing to determine the impact of BIB on the gut microbial composition and environment, and pinpoint the association between the microbial rearrangement and variations in the host phenotype.

## Materials and methods

### Patients

Twenty severely obese patients were evaluated for bariatric surgery. Eleven subjects were eligible for BIB, by fulfilling the following criteria: (1) morbid obesity [body mass index (BMI; in kg m^−2^) > 40] or severe obesity (BMI > 35), at least one comorbidity factor (arterial hypertension, diabetes mellitus) for ≥ 5 years, and resistance to medical treatment; (2) absence of medical or psychological contraindications for bariatric surgery; (3) absence of current excessive drinking, as defined by an average daily consumption of alcohol of 20 g d^−1^ for women and 30 g d^−1^ for men, and no history of excessive drinking for a period > 2 years at any time in the past 20 years; (4) absence of long-term consumption of hepatotoxic drugs; and (5) negative screening for chronic liver diseases, including hepatitis B surface antigen and hepatitis C virus antibodies, and other known liver diseases such as Wilson's disease, hemochromatosis, α1-antitrypsin deficiency, or autoimmune hepatitis; and (6) no malignant diseases. Informed written consent was obtained from all patients, and the study was conducted in conformity with the Helsinki Declaration. The Ethics Committee of the University Hospital Second University of Naples, Naples (Italy) approved the study. At baseline, all patients were evaluated by a multidisciplinary team consisting of dieticians, gastroenterologists, psychiatrists, an anesthesiologist, a cardiologist, an endocrinologist, and surgeons. The main demographic characteristics of patients are reported in Table 1.

**Table 1:** **Baseline demographic and biochemical characteristics of the patients studied**.

	**Patients**										
	**1**	**2**	**3**	**4**	**5**	**6**	**7**	**8**	**9**	**10**	**11**
Age (years)	45	56	46	50	48	56	64	58	35	58	41
Sex (F/M)	F	F	F	M	F	M	F	F	F	F	F
Body weight (kg)	102.40	80.60	125.00	107.00	73.40	113.00	115.00	101.90	110.00	103.00	145.00
BMI (kg m^−2^)	45.90	42.49	51.42	39.21	33.37	44.08	53.91	46.65	53.80	52.41	58.82
Diabetes mellitus (Y/N)	Y	N	Y	Y	Y	N	Y	N	N	Y	N
Arterial hypertension (Y/N)	Y	Y	Y	N	Y	Y	N	N	Y	Y	N
Insulinemia (mIU L^−1^)	16.00	17.00	11.80	24.00	16.00	31.60	11.80	15.00	18.00	21.00	21.90
Glycemia (mg dL^−1^)	117.00	83.00	129.00	109.00	130.00	99.00	129.00	98.00	92.00	107.00	99.00
ALT (IU L^−1^)	48.00	61.00	46.00	85.00	54.00	56.00	40.00	78.00	26.00	89.00	64.00
yGT (IU L^−1^)	38.00	66.00	64.00	54.00	49.00	94.00	84.00	42.00	51.00	43.00	89.00
Total cholesterol (mg dL^−1^)	179.00	254.00	163.00	154.00	179.00	265.00	163.00	216.00	190.00	237.00	214.00
Triglycerides (mg dL^−1^)	75.00	188.00	108.00	128.00	202.00	148.00	108.00	101.00	92.00	93.00	141.00
Caloric intake (kcal d^−1^)	3825.50	2505.40	3297.79	3511.70	3379.00	3809.10	1949.20	3468.21	1410.10	3446.90	2486.30

F, female; M, male; Y, yes; N, no; BMI, body mass index; ALT, alanine aminotransferases; γGT, gamma-glutamyl transpeptidase; IU, international units.

### Biochemical evaluation

Subjects fasted overnight for at least 12 h before an antecubital vein catheter was inserted for blood collection. The following clinical and biological features were assessed before surgery: BMI, alanine aminotransferases (ALT), gamma-glutamyl transpeptidase (γGT), serum triglycerides, cholesterolemia, fasting blood glucose, and fasting insulin. The energy intake from diet was obtained by recording individual dietary habits using the Winfood Software 2.0 package (Medimatica s. r. l., Martinsicuro, Italy) program, which calculates calories on the basis of the quantities and qualities of consumed food (Musso et al., 2005). Biochemical characteristics of patients at baseline are reported in Table 1. Parameters related to weight loss, glucose, and lipid metabolism were also evaluated 6 months after bariatric surgery.

### DNA extraction

Fecal samples were collected before and 6 months after BIB. Fifty milli gram of feces were homogenized in 500 μl of 10 mg/ml lysozyme solution in Tris-sucrose buffer (50 mM Tris-HCl, 40 mM EDTA, 0.75 M sucrose, pH 8.0) and incubated for 1 h at 37°C. The DNA was purified using the Maxwell® 16 DNA Purification Kit and the Maxwell® 16 Instrument (Promega, Madison, WI, USA), according to the manufacturer's instructions. The DNA quality was checked by agarose gel electrophoresis and the DNA concentration was determined using the Qubit HS dsDNA fluorescence assay (Life Technologies, Carlsbad, CA, USA).

### Illumina sequencing of the 16S rRNA gene

The V4 region of the bacterial 16S rRNA gene was PCR amplified using the primers 520F and 802R, which have a high overall coverage and reasonably good domain specificity (Klindworth et al., 2013). To allow multiplexing during Illumina sequencing, both forward and reverse primers were modified by the addition of a seven base long tag. For each sample the DNA amplification was performed in triplicate using 5 ng of DNA for each reaction. The PCR conditions were previously reported (Claesson et al., 2009). The resulting amplicons were checked by capillary electrophoresis using a QIAxcel (Qiagen GmbH, Hilden, Germany). All amplicons were purified using the MinElute PCR purification kit (Qiagen GmbH, Hilden, Germany) and then mixed together in equimolar concentrations. The library was sequenced on a MiSeq System using the MiSeq Reagent Kit v3 at a 2 × 250 bp read length configuration Paired-end sequencing (2 × 250 bp) was carried out on an Illumina MiSeq sequencer (Fasteris, Genève, Switzerland).The OBITools programs (www.prabi.grenoble.fr/trac/OBITools) were used to analyze the DNA sequence reads from Illumina, including assembling pair-end reads, assigning each read to its original sample, and de-replicating reads into unique sequences. To denoise the sequence dataset, singletons, and short sequences (< 20 nucleotides) were first removed using the obigrep command and then the sequences were cleaned of PCR/sequencing errors and chimeras using the obiclean program. For taxonomic assignment of sequences, a reference database was built using the ecoPCR program, which performs an in silico PCR to extract all sequences from the EMBL nucleotide library that may be amplified by the 520F/802R primer pair. The taxons were assigned using the EcoTag program. To further eliminate putative PCR and/or sequencer artifacts, bioinformatic filtering was used and only sequences with a 90% identity to the sequences present in GenBank were kept for further analysis.

### Real-time PCR

To determine the presence of hydrogen consumers and butyrate producers, PCR was performed on fecal DNA using the primer sets listed in Table 2. The qPCR assays were run using the StepOne™ Plus Real-Time PCR System (Applied Biosystems, Foster City, CA, USA). The amplification reaction was carried out in 20 μl containing 2 μl DNA, 0.4 mM of each forward and reverse primer, and 10 μl of KAPA SYBR FAST qPCR Master Mix (Kapa Biosystems, Wilmington, MA, USA). Standard curves were determined using plasmids containing amplified fragments of *acs, dsrA, mcrA*, or genomic DNA from reference strains for *BcoAT*. Results were expressed as gene copy numbers per gram wet feces.

**Table 2:** **Real-time PCR primers used to amplify microbial functional genes**.

**Primers**	**Target Gene**	**Functional Group**	**References**
ACS_f/ACS_r	*acsB*	Acetogens	Gagen et al., 2010
qmcrA-F/qmcrA-R	*mcrA*	Methanogens	Denman et al., 2007
DRS1F+/DSR-R	*dsrA*	Sulfate-reducing bacteria	Kondo et al., 2004
BcoAf/BcoAr	*BcoAT*	Butyrate-producing bacteria	Louis and Flint, 2007

acsB, acetyl-CoA synthase gene; mcrA, methyl-coenzyme M reductase gene; dsrA, dissimilatory (bi)sulfite reductase gene; BcoAT, butyryl-CoA: acetate CoA transferase gene.

### Metabolite analysis

Fecal samples (1.0 g weight) were diluted at a ratio 1:4 (w/v) in sterile distilled water. The pH of the fecal extracts was measured using a pH meter (GLP 21; Crison Instruments SA, Alella, Barcelona, Spain). The samples were then vortexed for 1 min and the homogenate centrifuged at 10,000 × g for 15 min at 10°C. The supernatant was transferred to a new tube and stored at −20°C until analysis. The Megazyme L-and D-Lactate Assay Kit (Megazyme International Ltd., Wicklow, Ireland) was used to detect D- and L-lactate. Urea was analyzed at 37°C via a clinical auto-analyzer (ILAB 600, Instrumentation Laboratory, Lexington, MA, USA) using a kit purchased from Instrumentation Laboratory (IL Test). An aliquot of fecal extract was acidified with 0.12 M oxalic acid and frozen at −20°C until the analysis of SCFAs. The SCFA concentration in fecal extract was analyzed by gas chromatography (model 7820A; Agilent Technologies, Santa Clara, CA, USA) using a capillary column (30 m × 250 μm × 0.25 μm; DB-FFAP, Agilent J&W GC column) and a flame-ionization detector. The oven temperature was held at 60°C for 5 min and then increased by 5°C/min to 170°C. The injector temperature was 250°C and the detector temperature was 300°C. The injector was equipped with a liner of glass wool to separate particles of dirt from the sample. The samples were injected by auto-sampler (1 μL volume) using the split method and a 25:1 splitting ratio. Hydrogen and air were used for flame ionization detection. The carrier gas was nitrogen, with a constant flow of 1.78 mL/min. Pivalic acid was used as an internal standard.

### Statistical analysis

To verify an adequate sequencing depth of the fecal microbiota, we performed rarefaction analysis on raw counts using an R-based protocol (http://www.jennajacobs.org/R/rarefaction.html). Alpha and beta diversity analysis was performed in R (version 3.1.2) (R Core Team, 2014) using the Vegan package (Oksanen et al., 2014). To avoid bias due to different sampling depths, we first rarefied the OTU abundance table by randomly subsampling each sample to the smallest data size. Differences between fecal bacterial communities were visualized using non-metric multidimensional scaling (NMDS) on the basis of the Bray-Curtis distance matrix. Hierarchical clustering using Spearman rank correlation distance and Ward linkage was performed on relative abundance values at the genus level using the hclust function of Vegan. To identify bacterial taxa that were differentially abundant in the gut microbiota of obese patients before and after surgery, we utilized the LEfSe algorithm (Segata et al., 2011) implemented in the Galaxy module (http://huttenhower.sph.harvard.edu/galaxy/). We used per-sample normalization on bacterial relative abundance data. The default value of alpha was 0.05 and the logarithmic LDA score threshold was 2. A principal component analysis (PCA) was carried out using the prcomp function in R to explore relationships between bacterial populations that significantly changed after BIB and clinical parameters of the host. To investigate associations among bacterial populations and SCFA concentrations, linear mixed-effects models were fitted by maximum likelihood (ML) with the lme function in the nlme R package (Pinheiro et al., 2013). After standardization to zero mean and unit variance, bacteria populations were tested as fixed independent effects, and SCFA concentrations as response variables. To take into account the repeated-measures design of the experiment, subjects were modeled as random factors. Likewise, associations between bacterial groups that significantly changed after BIB and anthropometric and biochemical measurements, including dietary caloric intake, were also assessed. The Bonferroni correction was used as an adjustment method for multiple testing, with a chosen significance level of *P* ≤ 0.05 for multiple comparisons of the associations between bacterial genera and SCFAs, and of *P* ≤ 0.20 for multiple comparisons between bacterial genera and bio-clinical markers. To compare population means for statistical significance, the Shapiro-Wilks test was used to check for normality, and paired *t*-test or Wilcoxon rank-sum tests were performed using either R or GraphPad Prism version 5 (Graphpad Software, San Diego, CA, USA).

### Nucleotide sequence accession

The 16S sequence data are publicly available on Dryad repository (http://dx.doi.org/10.5061/dryad.j82n2).

## Results

### Bio-clinical characteristics of surgical patients

As expected, obese individuals showed a significant reduction in BMI and body weight 6 months after surgical intervention (Table 3). Circulating glucose and insulin were also decreased, which indicated an amelioration of glucose homeostasis, as well as postoperative triglyceride and total cholesterol concentrations. An analysis of total food intake showed a drastic caloric reduction 6 months after surgery compared with the pre-operative nutritional status (Table 3).

**Table 3:** **Characteristics of the study population pre- (baseline) and post-surgery (6 months after bilio-intestinal bypass)**.

**Parameters**	**Pre-Surgery (*n* = 11)**	**Post-Surgery (*n* = 11)**
Body weight, kg	106.94±19.41	89.21±17.90 ^***^
BMI, kg m^−2^	47.46±7.46	40.68±5.88 ^***^
Insulin, mIU L^−1^	18.55±5.80	13.55±2.16 ^**^
Glucose, mg dL^−1^	108.36±16.10	93.36±10.93 ^***^
Cholesterol, mg dL^−1^	201.27±38.45	152.09±33.27 ^***^
Triglycerides, mg dL^−1^	125.82±40.56	102.00±35.32 ^**^
Caloric intake, kcal d^−1^	3008.11±799.31	1540.28±378.16^***^

BMI, body mass index. Values are the mean ± SD (n = 11). Paired Student's t-test was performed to compare values before and after bilio-intestinal bypass. Asterisks indicate statistical significance (

** P < 0.01;

*** P < 0.001).

### Sequencing coverage and estimation of fecal bacterial diversity

**Figure 1:**
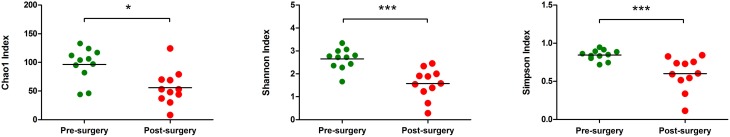
**Alpha diversity metrics (Chao 1, Shannon and Simpson indices) of OTU-level fecal bacterial communities from obese individuals; each point represents a sample**. Paired Student's *t*-test was performed to compare alpha diversity estimates before and after bilio-intestinal bypass. Asterisks indicate statistical significance (^*^P < 0.05; ^***^P < 0.001).

**Figure 2:**
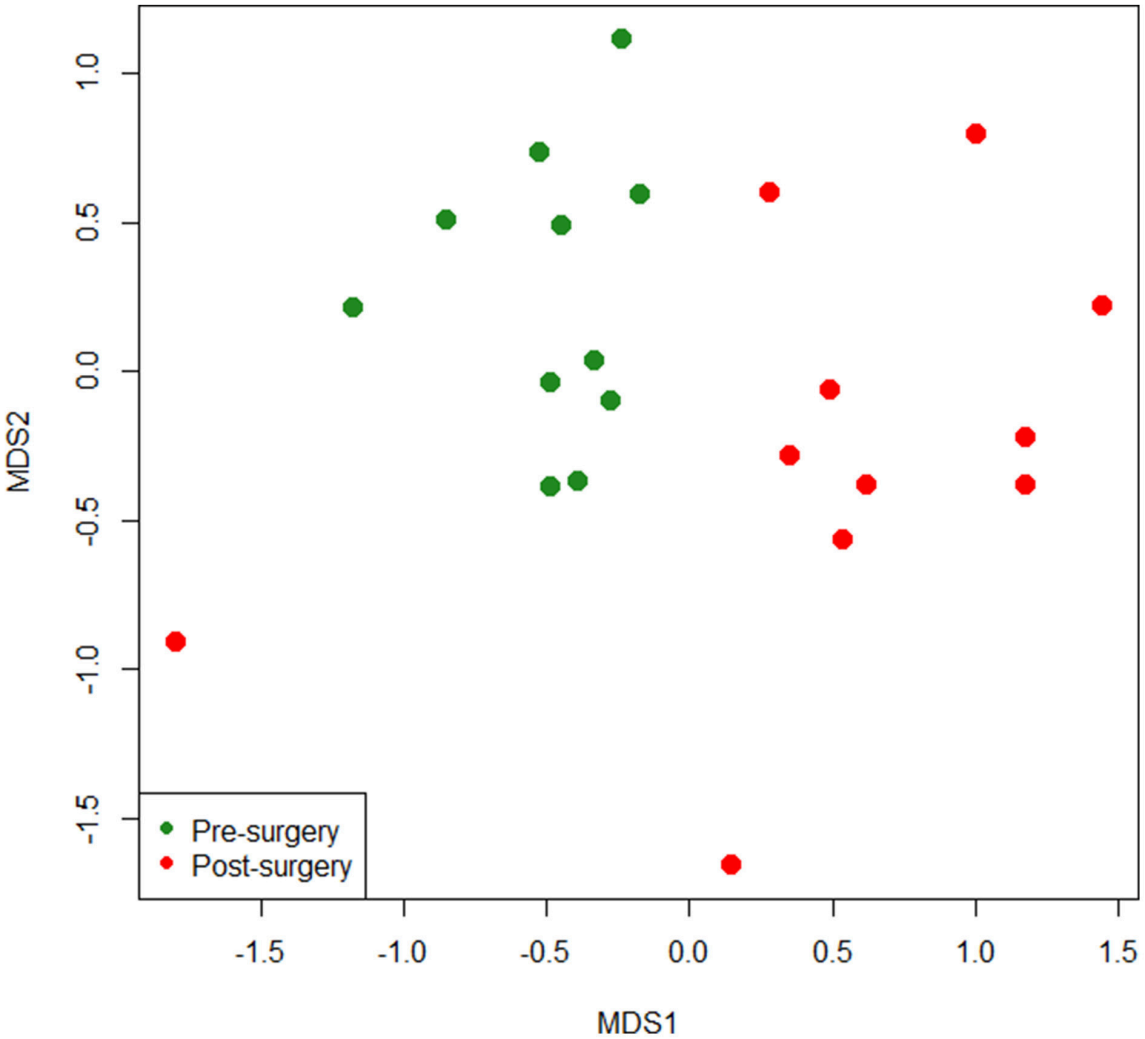
**Non-metric multidimensional scaling (NMDS) ordination plot based on Bray-Curtis dissimilarities between OTU-level fecal bacterial communities in obese individuals before and after bilio-intestinal bypass**.

In this study, the bacterial composition of the fecal samples was examined using an Illumina high-throughput sequencing technique. We generated a dataset consisting of 1,232,275 filtered high-quality and classifiable 16S rRNA gene sequences, and the average number of sequences obtained for each individual was 56,013 (range: from 25,224 to 102,372). All sequences were clustered with representative sequences, and a 97% sequence identity cut-off was used. The total number of operational taxonomic units (OTUs) obtained was 1244. Rarefaction curves showed that, for each subject, bacterial species richness leveled off as sampling depth increased, indicating that the sequencing effort was sufficient and that the total diversity within the sample was captured (Supplementary Figure 1). They also suggested that the alpha diversity in pre-surgery samples was higher than in post-surgery samples. To confirm this observation, Chao1 indexes were calculated and evaluated by paired *t*-test (Figure 1). There was a significant decrease in the mean microbial richness in post-surgery subjects compared with pre-surgery (*P* = 0.018). A diversity analysis based on Simpson and Shannon indexes revealed that the diversity of the intestinal microbiota at the OTU level was significantly higher in the fecal samples of pre-surgery subjects compared with post-surgery (*P* = 0.0009 and *P* = 0.0008, respectively). To compare the composition of the intestinal microbiota of patients before and after surgery, non-metric multi-dimensional scaling (NMDS) of a Bray-Curtis distance matrix based on the abundance of OTUs was employed. The NMDS revealed differences in the microbiota composition based on surgery (Figure 2). An apparent clustering pattern was identified for the participants before and after BIB. Points representing the intestinal microbiota composition of obese subjects and treated subjects clustered at the top left and the top right, respectively.

### Taxonomic composition of fecal bacterial communities

**Figure 3:**
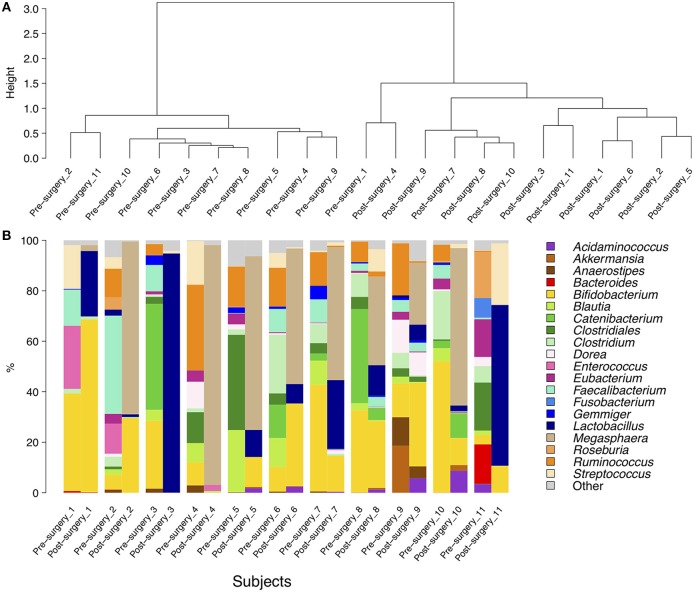
**Hierarchical clustering (A) and a barplot of relative abundances (B) of bacterial genera found within the fecal communities of obese patients**. Only genera with a relative abundance ≥5% in at least one subject are represented. The dendrograms were calculated using Spearman's rank correlation and Ward-linkage clustering.

Firmicutes, Actinobacteria, Bacteroidetes, Proteobacteria, and Verrucomicrobia constituted the five dominant bacterial phyla in the obese subjects under study, making up an average of 75.59, 21.62, 0.89, 0.82, and 1.05% of the total sequences, respectively (Supplementary Figure 2). At the family level, the predominant taxa were Veillonellaceae, Bifidobacteriaceae, Ruminococcaceae, Lactobacillaceae, Lachnospiraceae, Erysipelotrichaceae, and Clostridiaceae (data not shown). A portion (4.4%) of sequences belonging to the order Clostridiales was not assigned to any family. At the genus level, reads were assigned to 73 genera. A barplot representation of genus-level sequence data showed high inter-individual variability in the bacterial profile, indicating that relative abundances of each genus varied a lot between individuals, both before and after intervention (Figure 3B). The most abundant genus in the gut of obese individuals was *Bifidobacterium*, which accounted for 22.2% of the total number of sequences in both pre- and post-surgery samples. *Lactobacillus* was the second most abundant genus in post-surgery individuals whereas *Roseburia* was the second most abundant genus in pre-surgery samples. *Megasphaera* was detected only in post-surgery individuals where it represented 16.1% of the sequences. Hierarchical clustering of the microbiota community composition at the genus level showed that almost all post-surgery profiles clustered separately from their pre-surgery counterpart (Figure 3A).

### Supervised comparison of the gut microbiota between pre- and post-surgery patients

**Figure 4:**
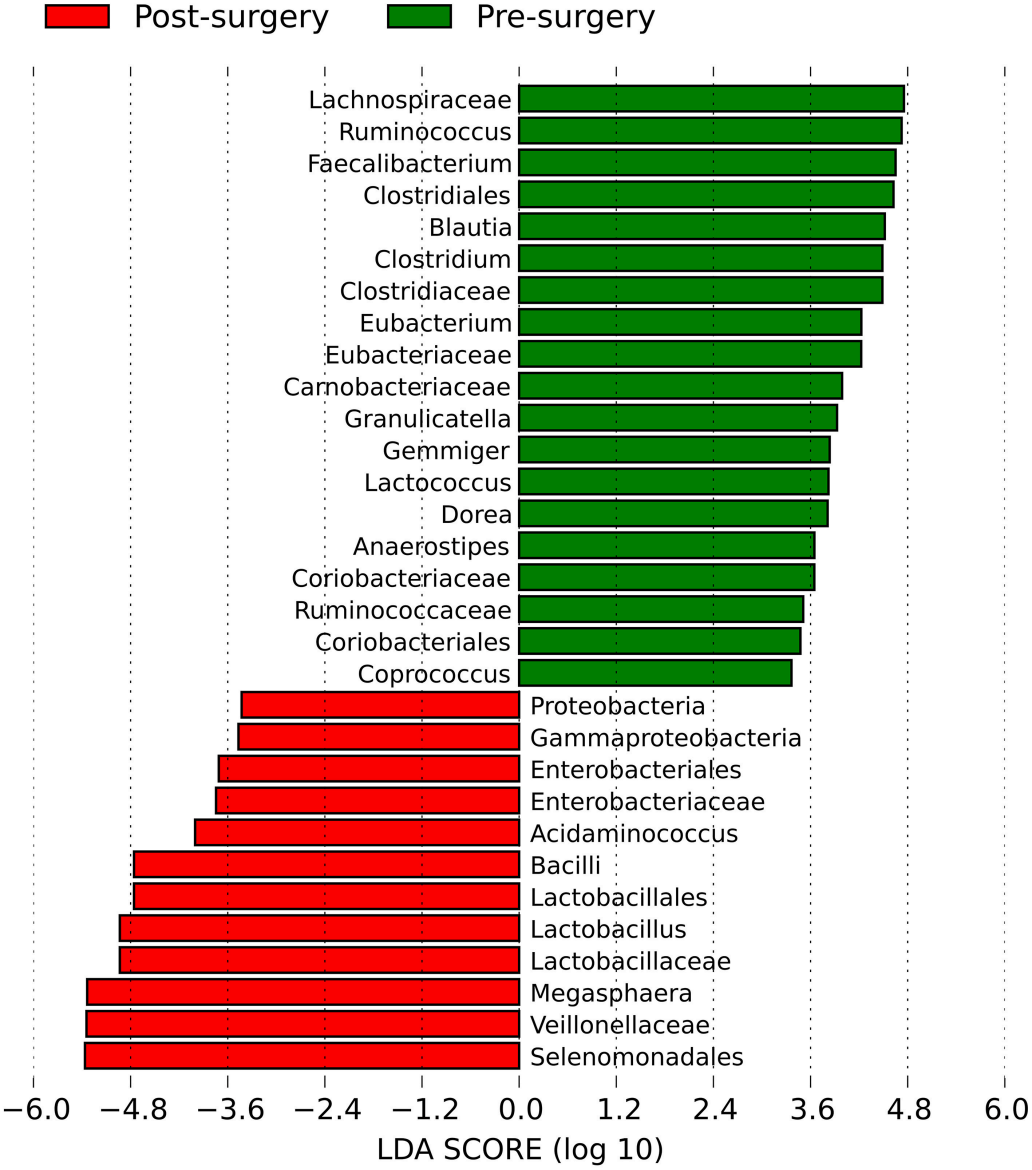
**Histogram of the linear discriminant analysis (LDA) scores for differentially abundant bacterial clades in fecal samples between obese individuals before and after bilio-intestinal bypass**. Negative (red bars) LDA scores represent bacterial groups over-abundant in post-surgery samples while positive (green bars) represent bacterial groups overrepresented in pre-surgery samples.

Some of these microbial groups were present in different concentrations in the feces of pre- vs. post-surgery individuals; therefore, we attempted to highlight genera showing significant differences. The linear discriminant analysis effect size (LefSE) method identified a total of 31 bacterial groups that were differentially abundant in obese individuals before and after bariatric surgery (Figure 4 and Supplementary Figure 3). There were 19 groups of bacteria enriched in the fecal samples of pre-surgery individuals, namely, Clostridiales (the order and its families Lachnospiraceae, Ruminococcaceae, Clostridiaceae, and Eubacteriaceae); Coriobacteriales (the order and its family Coriobacteriaceae); Carnobacteriaceae (the family and its genus *Granulicatella*); and *Lactococcus*. Within Clostridiales, genera with a linear discriminant analysis (LDA) score ≥4 were *Faecalibacterium* (*P* = 0.0019), *Ruminococcus* (*P* = 0.0007), *Clostridium* (*P* = 0.0001), *Eubacterium* (*P* = 0.0007), and *Blautia* (*P* = 0.0003). The bacterial groups enriched in post-surgery patients were Selenomonadales (the order, its family Veillonellaceae, and the two genera *Megasphaera* and *Acidaminococcus*), the family Enterobacteriaceae (from phylum Proteobacteria to order Enterobacteriales), and the genus *Lactobacillus* (from the class Bacilli to the family Lactobacillaceae). Within these, genus *Megasphaera* had the highest LDA score of 5.33 (*P* = 0.0002), followed by *Lactobacillus* with a LDA of 4.93 (*P* = 0.0009) and *Acidaminococcus* with a LDA of 4.00 (*P* = 0.035).

### Associations between key bacterial populations and host parameters

Principal component analysis (PCA) (Supplementary Figure 4) indicated that some putative correlations could exist between significant bacterial populations and host clinical parameters. Univariate linear mixed models were then used to substantiate such relationships. Analysis revealed several significant associations but, after adjusting for multiple testing, only five models showed a *P* ≤ 0.20. Among them, positive associations were found between *Clostridium* levels and blood insulin (*B* = 0.728, *P* = 0.019; where *B* is the estimated standardized regression coefficient and *P* the related *P*-value after Bonferroni correction), *Faecalibacterium* and triglycerides (*B* = 0.610, *P* = 0.002), *Gemmiger* and serum glucose (*B* = 0.631, *P* = 0.053), and total cholesterol and *Clostridium* (*B* = 0.696, *P* = 0.141). There was a negative relationship between blood glucose and the relative abundance of *Lactobacillus* (*B* = -0.551, *P* = 0.141). To evaluate if such associations were influenced by variations in dietary calories, bivariate linear mixed models were fitted including caloric intake as a fixed effect. Only models in which the collinearity among predictors was not significant were run. Among these, associations between glucose and *Gemmiger* (*B* = 0.469, *P* = 0.0006), glucose and *Lactobacillus* (*B* = −0.385, *P* = 0.0065), and triglycerides and *Faecalibacterium* (*B* = 0.592, *P* = 0.0002) remained significant even when controlling for caloric intake. In the first two aforementioned models, caloric intake displayed a significant positive association with glucose (*B* = 0.360 and 0.347, respectively, with associated *P* = 0.0021 and 0.0079, respectively), thus showing a simultaneous independent effect on blood glucose concentration; while, there was no significant association in the last model (*B* = 0.035, *P* = 0.7066).

### Quantification of functional bacterial groups

**Figure 5:**
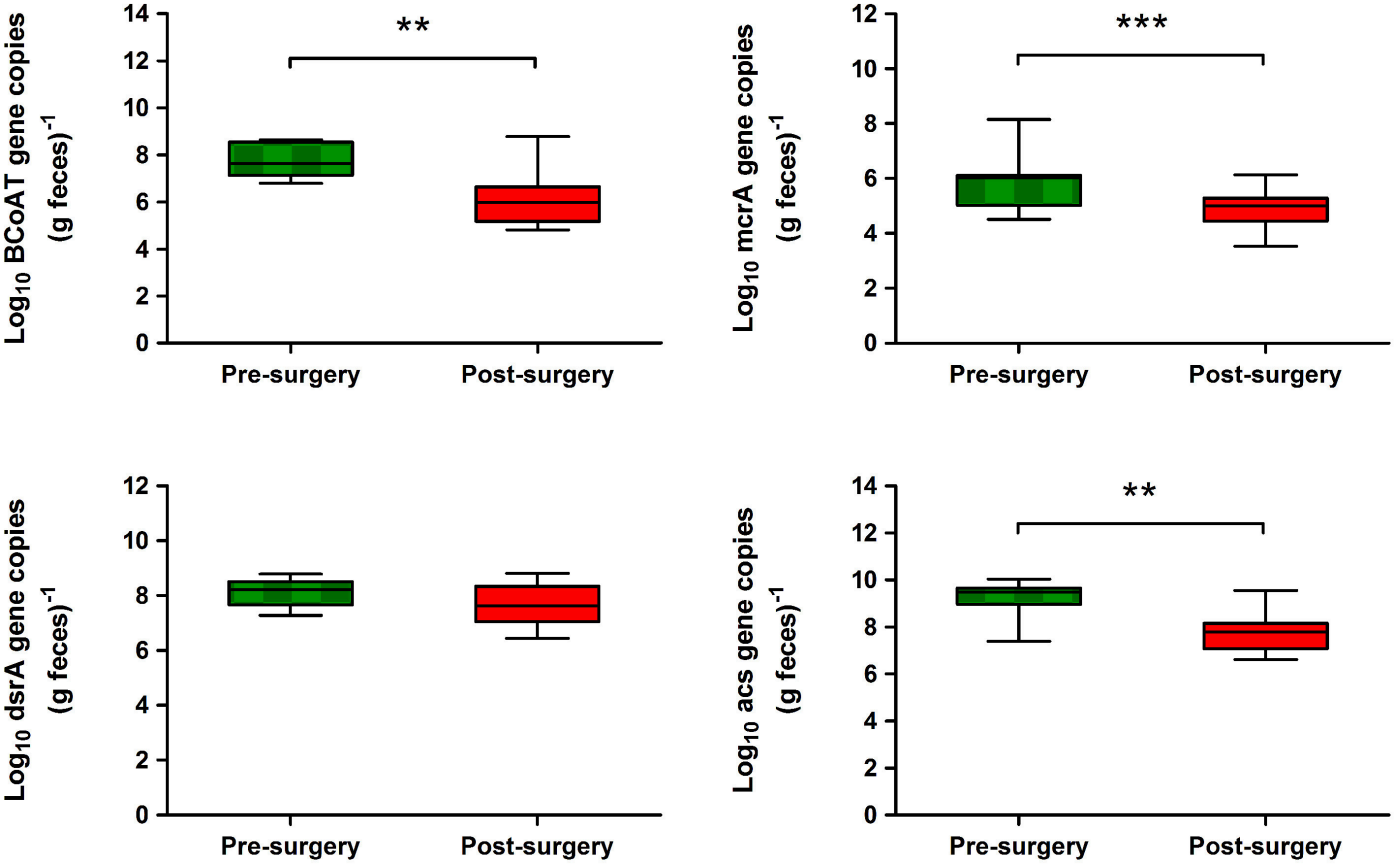
**Abundance of metabolic functional genes in the fecal microbiota of obese individuals before and after bariatric surgery as determined by qRT–PCR**. Genes targeted included *BcoAT* for butyrate producers, *acs* for acetogens, *mcrA* for methanogens, and *dsrA* for sulfate reducers. Values are log means ± SD. Paired Student's *t*-test was performed on log-transformed data. Asterisks indicate statistical significance (^**^P < 0.01; ^***^P < 0.001).

Real-time PCR analysis was performed to detect bacterial functional groups whose metabolic activities play a key role in human colon metabolism. The qRT–PCR of the butyryl-CoA:acetate CoA-transferase (*BCoAT*) gene, involved in the microbial production of butyrate, indicated that the average abundance of the *BcoAT* gene was significantly reduced by about 2 log in post-surgery subjects compared with pre-surgery (7.68 ± 0.69 vs. 6.05 ± 1.15 log_10_ gene copies g^−1^, *P* = 0.0048) (Figure 5). Mean acetogenic bacteria levels were significantly lower in post-surgery subjects than in pre-surgery individuals (7.68 ± 0.85 vs. 9.21 ± 0.73 log_10_ gene copies g^−1^, *P* = 0.0019) (Figure 5). Similarly, there was a significant post-surgery reduction in the mean number of methanogens (4.93 ± 0.70 log_10_ gene copies g^−1^) compared with baseline (5.95 ± 1.01 log_10_ gene copies g^−1^, *P* = 0.001). In contrast, quantitative PCR of the dissimilatory (bi)sulfite reductase gene revealed that mean levels of sulfate-reducing bacteria were similar in the two groups (Figure 5).

### Bacterial metabolism products in fecal samples

**Figure 6:**
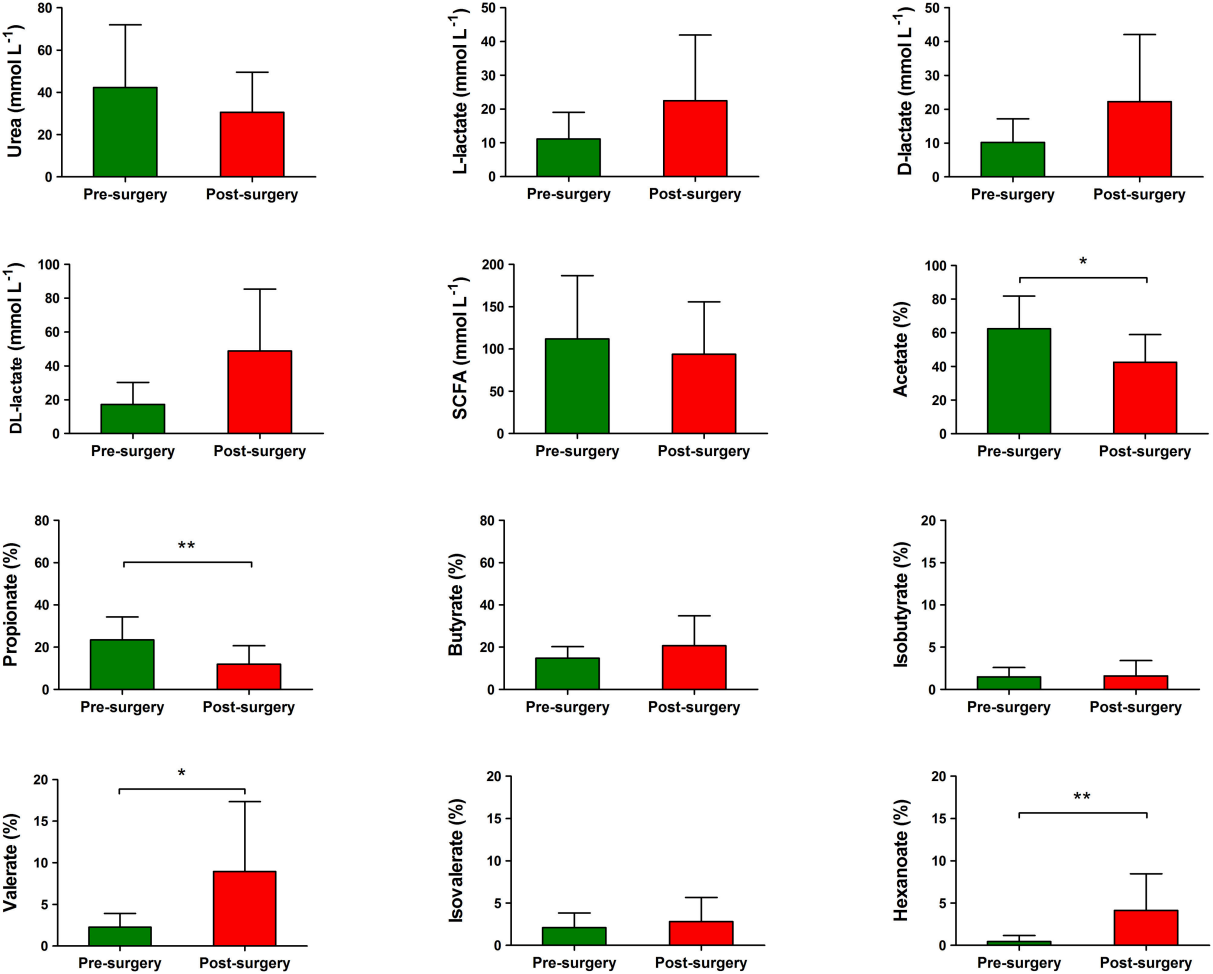
**Levels of bacterial metabolites in the fecal samples of obese individuals before and after bilio-intestinal bypass. Individual short chain fatty acids (SCFAs) are shown as mean percentages of the total SCFA concentration**. Values are means ± SD. Wilcoxon signed-rank test was performed to compare metabolites values before and after bilio-intestinal bypass. Asterisks indicate statistical significance (^*^P < 0.05; ^**^P < 0.01).

The pH of fecal samples was significantly lower in post-surgery subjects compared with pre-surgery (5.72 ± 0.70 vs. 6.74 ± 0.74, *P* = 0.008). No statistically significant difference was found between the mean levels of urea in fecal samples from subjects pre-surgery compared with post-surgery (Figure 6). Both L-lactate and D-lactate increased in post-surgery subjects; although, such differences did not reach statistical significance. Relative to pre-surgery samples, there were significantly lower levels of acetate (42.63 ± 16.34 vs. 62.40 ± 19.39% of total short chain fatty acids (SCFA), *P* = 0.0117) and propionate (11.94 ± 8.750 vs. 23.42 ± 10.95% of total SCFA, *P* = 0.0049) in the post-surgery samples. On the contrary, the fecal samples from post-surgery individuals had significantly higher levels of valerate (8.956 ± 8.390 vs. 2.258 ± 1.660% of total SCFA, *P* = 0.0162) and hexanoate (4.117 ± 4.364 vs. 0.4600 ± 0.7202% of total SCFA, *P* = 0.0039) compared with pre-surgery samples (Figure 6). The fecal content of butyrate did not significantly vary in post-surgery patients compared with pre-surgery. When we tested for associations between the relative abundance of bacterial genera and fecal metabolite parameters, we observed significant positive relationships between hexanoate percentage levels and *Megasphaera* (*B* = 0.899, *P* = 0.0008) and valerate percentage levels and *Megasphaera* (*B* = 0.773, *P* = 0.04).

## Discussion

In our study, obese patients had high mean levels of Firmicutes and low levels of Bacteroidetes in their fecal samples, when evaluated by Illumina 16S rDNA sequencing. Several authors have pointed out that human obesity is associated with high gut concentrations of Firmicutes and low concentrations of Bacteroidetes compared with lean individuals (Ley et al., 2006; Turnbaugh et al., 2009). This finding was not supported by other studies (Duncan et al., 2008; Schwiertz et al., 2010) and a recent analysis of data retrieved from the Human Microbiome Project (HMP) and MetaHIT survey showed no association between obesity and the Bacteroidetes-to-Firmicutes ratio (Finucane et al., 2014). Notably, there are reports of reduced Firmicutes and increased Bacteroidetes in obese subjects after weight loss induced by either gastric bypass (Zhang et al., 2009; Kong et al., 2013) or dietary restriction (Ley et al., 2006; Duncan et al., 2008). In this study, we did not observe either a decrease in Firmicutes abundance or an increase in Bacteroidetes in BIB-treated patients after surgery, i.e., the Bacteroidetes and Firmicutes bacterial populations shifting to proportions more like those usually found in lean individuals. Interestingly, the amounts of fecal Bacteroidetes detected in our study population were very much lower than those previously estimated in human obese individuals. The source of this discrepancy is unknown, but potential factors influencing the observed pattern include obesity grade, comorbid conditions, energy intake, age, population size, geographical location.

At lower taxonomic rank, we found several bacterial features that most likely explain the differences between pre- and post-surgery fecal microbiota. The bacterial genera within the Firmicutes phylum that were abundant in obese individuals included mostly members of the order Clostridiales. Several authors have reported enrichment of *Clostridium* cluster XIVa (Verdam et al., 2013), *Roseburia intestinalis* (Tims et al., 2013; Verdam et al., 2013), and *Eubacterium rectale* (Ferrer et al., 2013; Tims et al., 2013) in the gut of obese subjects. In addition, pre-surgery obese individuals had increased Coriobacteriaceae. Members of the family Coriobacteriaceae represent dominant gut bacteria that are not well studied; although, they have important metabolic functions such as the conversion of bile salts and steroids that may influence host lipid metabolism. In support of this, studies in rodents determined that the abundance of this bacterial group significantly correlated with triglyceride levels or non-HDL cholesterol levels (Martínez et al., 2009; Claus et al., 2011). In post-surgery patients, the LefSE method showed a significant increase in Proteobacteria (Gammaproteobacteria), specifically Enterobacteriaceae, compared with pre-surgery. This result is in agreement with previous studies on the effects of Roux-en-Y gastric bypass on gut microbiota that showed a substantial shift toward higher concentrations of Proteobacteria after surgery in both humans and rats (Zhang et al., 2009; Li et al., 2011; Graessler et al., 2013; Kong et al., 2013). In addition, the fecal microbiota of post-surgery patients was significantly enriched in bacterial taxa belonging to the order Lactobacillales and Selenomonadales, in particular the genera *Lactobacillus, Acidaminococcus*, and *Megasphaera*. The *Lactobacillus* spp. produce lactic acid and derive energy from the fermentation of lactose, glucose, and other sugars; while, *Acidaminococcus* spp. are among the predominant amino acid-fermenting microbiota along the digestive tract of humans and animals. Glucose and amino acid absorption mainly take place in the small intestine, so it is tempting to speculate that small intestinal resection, by increasing the amount of simple sugars and amino acids reaching the large intestine, may trigger colon bacteria that can use mal-absorbed nutrients as an energy source. This mechanism has been described in human short bowel syndrome, where fermentation of non-digested carbohydrates in the large bowel can lead to a decrease in luminal pH as a result of the overgrowth of D-lactate producing bacteria, such as *Lactobacillus* spp. (Buchman et al., 2003; Zhang et al., 2003). Consistent with this, we observed a significant decrease of pH in fecal samples from post-surgery individuals, together with an increase in lactic acid, although the latter did not reach statistical significance. It seems also likely that an increased activity of lactate-producing bacteria in the distal gut may be linked to the augmented numbers of *Megasphaera* after surgery. *Megasphaera elsdenii*, the most studied of the *Megasphaera* spp., is known to actively ferment lactate in the rumen, thereby reducing acidosis in ruminants (Marounek et al., 1989). It has been suggested that *Megasphaera* spp. isolated from the human gut may serve a similar function (Shetty et al., 2013).

To gain further insight into the microbial community shifts associated with BIB, we evaluated the abundances of functional bacterial groups. We detected a significant decrease in the *BCoAT* gene post-surgery. This result is not unexpected, since production of butyrate through BCoAT appears to be a common underlying feature of bacteria within the class Clostridia (Louis et al., 2010). Among hydrogenotrophic gut bacteria, a significant decrease of acetogenic bacteria was found after BIB. These results are in line with data from Illumina sequencing of 16S rRNA gene amplicons, in that several bacterial groups that were significantly reduced in stool from post-surgery patients, including the genera *Ruminococcus, Clostridium*, and *Blautia*, are known acetate producers (Chassard and Bernalier-Donadille, 2006). Analogously, we found a reduction of methanogenic Archaea after bariatric surgery using quantitative PCR with *mcrA* primers. These findings confirm results from Furet et al. (2010) and Zhang et al. (2009). Our results from acetogenic and methanogenic populations seem to further support the hypothesis of Zhang et al. (2009) that, in obese individuals, methanogens, and likely acetogens, accelerate the fermentation of indigestible polysaccharides by lowering the partial pressure of hydrogen. Thus, the energy harvesting from the diet is increased. Notably, we did not find any significant changes in the levels of sulfate-reducing bacteria in post-surgery patients compared with pre-surgery. One explanation for this is that the metabolism of sulfate-reducing bacteria is much more versatile than that of acetogens and methanogens since they can oxidize a variety of organic compounds such as lactate (Marquet et al., 2009) and can persist under different environmental conditions (Muyzer and Stams, 2008).

The observed shifts in bacterial populations between obese subjects before and after BIB led us to investigate potential changes in the gut environment conditions and fermentation parameters. The post-surgical decrease of pH points toward an altered intestinal environment that could reasonably influence gut microbial ecology, as indicated previously (Walker et al., 2005). Studies in animal models have shown that the obese microbiota differs from the lean microbiota and may produce more SCFAs and, hence, extract more energy from a given diet than the lean microbiota (Turnbaugh et al., 2006). Recent studies have also shown that fecal SCFA concentrations were significantly higher in obese subjects compared with lean participants (Schwiertz et al., 2010). However, in our study, we did not detect any significant variations in the mean levels of total SCFAs and butyrate in obese individuals before and after bariatric surgery. Although, it must be stressed that a real-time PCR assay targeting the *BcoAT* gene indicated a decrease of butyrate producers in the gut of surgical patients. In a recent study conducted in swine, several isolated strains, including *M. elsdenii*, did not have *BcoAT* genes detectable by degenerate PCR assay; although, they all had *BcoAT* enzyme activity. Authors suggested that the degenerate primers for *BcoAT* genes could underestimate the abundance of butyrate-producing bacteria and should be broadened to capture more butyrate producers in the gut (Levine et al., 2013). In the study by Liou et al. (2013), the RYGB mice had decreased total SCFAs compared with sham mice, with reductions in acetate levels and a relative increase in propionate levels. In our obese patients, the ratios of individual SCFAs are altered, with decreased acetate and propionate as well as increased valerate and hexanoate fecal concentrations. Such compounds are related to gut bacterial metabolism; thus, it is likely that variations in the SCFA profile of post-surgery patients reflect the specific composition of the gut microbial community. When we attempted to find linear relationships between bacterial groups and metabolites, we found that hexanoate and valerate levels were significantly associated with the relative proportion of *Megasphaera* spp. This is not surprising, since the rumen bacterium *M. elsdenii* reportedly produces valerate and caproate from redox-neutral substrates (sugars or lactate) (Marounek et al., 1989). It is can be hypothesized that the final concentrations of butyrate, acetate, and propionate in feces are the result of not only shifts in the direct production by microbial populations, but also of the interspecies cross-feeding interactions occurring in the distal gut. Further studies are necessary to elucidate the potential contributions of the specific microbial-derived metabolite changes on host colonic function and energy balance after BIB. In this regard, butyrate is the major energy source among SCFAs for colonocytes; while, acetate and propionate are absorbed into portal circulation. Propionate is largely taken up by the liver and consumed for gluconeogenesis; however, acetate is metabolized by peripheral tissues for de novo lipogenesis (Wong et al., 2006). Jørgensen et al. (1997) suggested that valerate and caproate are excellent substrates for oxidation in rat colonocytes, similar to butyrate. In addition to being used as an energy source for the host, the signaling properties of SCFAs could indicate a putative contribution to the changes associated with patients before and after the intervention. The SCFAs are ligands for the G protein–coupled receptors Gpr41 and Gpr43. Acetate exhibits higher potency at the human GPR43, whereas valerate and caproate have higher potency at human GPR41 (Brown et al., 2003; Le Poul et al., 2003). Notably, a growing body of evidence links GPR43 to body weight and metabolic disorders (Hong et al., 2005; Ge et al., 2008; Bjursell et al., 2011). Data in several animal studies have highlighted a role of GPR41 in regulating host energy balance, in particular by acting on sympathetic activity and intestinal gluconeogenesis (Samuel et al., 2008; Kimura et al., 2011; De Vadder et al., 2014).

Despite the small size of the study cohort, we succeeded in highlighting a few significant associations between alterations in metabolic indices and changes in the composition of the gut microbiota. In particular, *Lactobacillus*, which was up-regulated after surgery, was negatively correlated with blood glucose. When looking at probiotic applications in humans and animal models, *Lactobacillus* spp. are effective in the regulation of glucose metabolism (Panwar et al., 2013). We found positive associations involving bacterial genera that decreased after BIB, such as *Clostridium* and blood insulin and total cholesterol, *Faecalibacterium* and triglycerides, and *Gemmiger* and blood glucose. Since dietary intake was significantly reduced after surgery, we verified such associations by controlling for the potential confounding effect of diet. After the statistical adjustment for caloric intake, these associations remained significant, indicating that the highlighted relationships between these bacterial populations (with the exception of *Clostridium*, which showed significant collinearity with caloric intake) and clinical parameters were independent of caloric intake. These results are in agreement with those of Kong et al. (2013) that found the populations of gut microbiota can be modulated via bariatric surgery independently of the variation in caloric consumption (Kong et al., 2013).

The present study has some limitations that have to be acknowledged. Obese patients experienced a marked decrease of body weight subsequent to the BIB as well as a significant reduction in caloric intake. The present sample size of 11 may be insufficient for highlighting every significant correlation pattern between gut microbiota and clinical parameters, and the observed correlations may be partly associated with other confounding variables. Regardless of the relevance of BIB for clinical practice, our study contributed to the identification of gut microbial populations and metabolite profiles that could represent putative markers of obesity, as well as markers of weight loss and metabolic improvement following bariatric surgery. These findings warrant further investigation to evaluate the exact impact of surgically-induced changes in human gut microbiota on host physiology and health. A deeper understanding of host-microbial crosstalk might help in the development of new alternative strategies as well as approaches to supplement bariatric surgery in the treatment of obesity and related co-morbidities.

## Author contributions

VP, AM, EV, ST, LD, AF, CL, and MD make substantial contributions to acquisition, analysis and interpretation of data. VP, AF, and AM participate in writing the article. MC, LM, and CL participate in critically revising the article.

### Conflict of interest statement

The authors declare that the research was conducted in the absence of any commercial or financial relationships that could be construed as a potential conflict of interest.

## Supplementary material

Supplementary Figure 1 **Normalized rarefaction curves displaying the average number of OTUs (at 97% similarity) discovered by random sampling within each fecal sample from obese subjects before (green line) and after (red line) bilio-intestinal bypass**.

Supplementary Figure 2 **Hierarchical clustering (A) and a barplot of relative abundances (B) of bacterial phyla found within the fecal communities of obese patients**. The dendrograms were calculated using Spearman's rank correlation and Ward-linkage clustering.

Supplementary Figure 3 **Cladogram indicating the phylogenetic distribution of differentially abundant bacterial lineages in fecal samples between obese individuals before and after bilio-intestinal bypass, determined by linear discriminant analysis (LDA)**. Red dots represent bacterial groups over-abundant in post-surgery samples, while green dots represent bacterial groups overrepresented in pre-surgery samples.

Supplementary Figure 4 **Biplot of the first two principal components from the principal component analysis (PCA) of bacterial genera relative abundances and bio-clinical markers**. The length of the vectors represents the PCA loadings of the variables on the first two principal components. Dots represent individual subjects before (solid circles) and after (open circles) bilio-intestinal bypass.
