# Supplementary figures and images for: Postoperative Changes in Fecal Bacterial Communities and Fermentation Products in Obese Patients Undergoing Bilio-Intestinal Bypass
Source: Front Microbiol. 2016 Feb 23;7:200. doi: 10.3389/fmicb.2016.00200 (PMC4762995; doi:10.3389/fmicb.2016.00200)

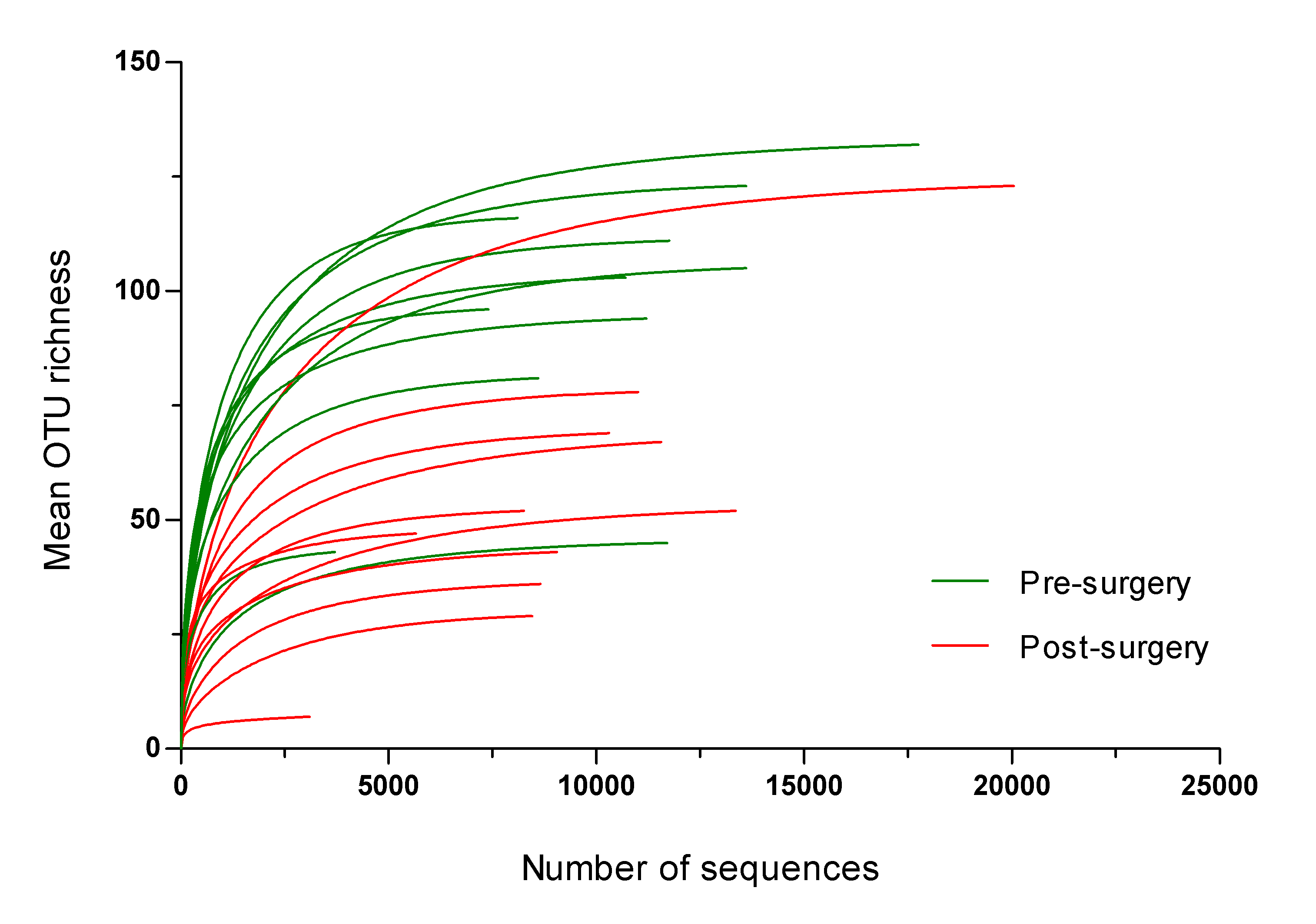

Supplement: Supplementary Figure 1 — Normalized rarefaction curves displaying the average number of OTUs (at 97% similarity) discovered by random sampling within each fecal sample from obese subjects before (green line) and after (red line) bilio-intestinal bypass. [file Image1.TIF]

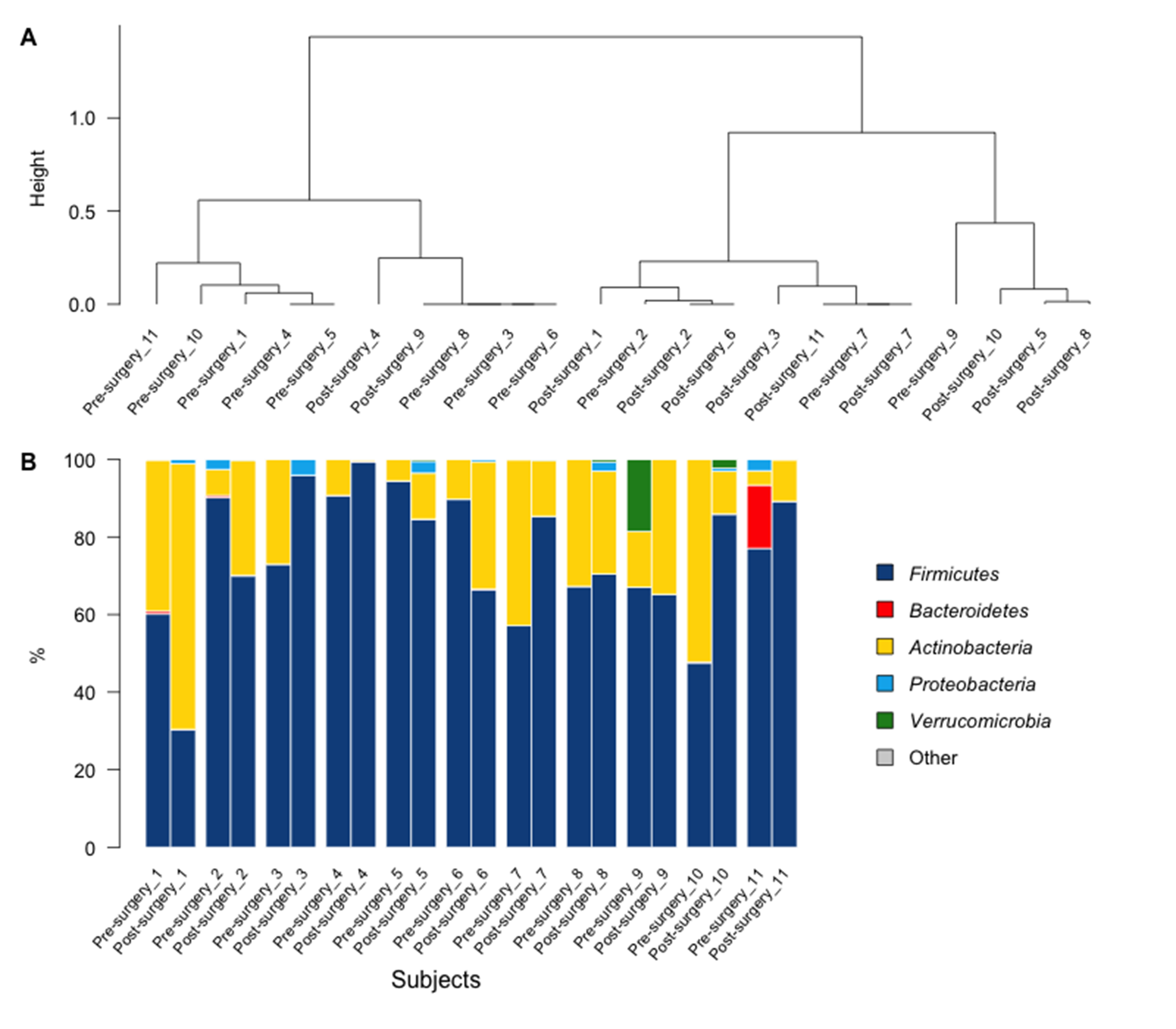

Supplement: Supplementary Figure 2 — Hierarchical clustering (A) and a barplot of relative abundances (B) of bacterial phyla found within the fecal communities of obese patients. The dendrograms were calculated using Spearman's rank correlation and Ward-linkage clustering. [file Image2.TIF]

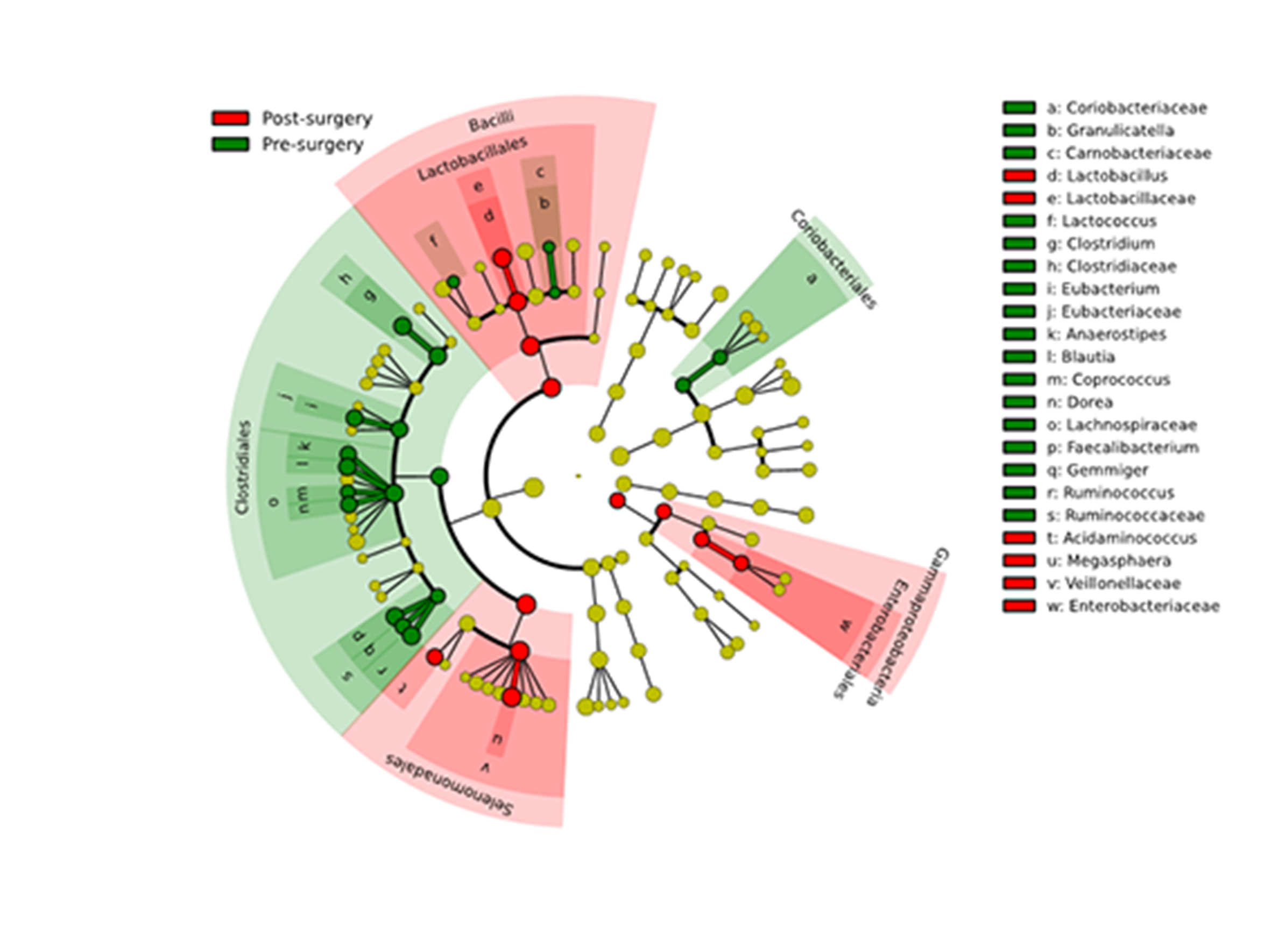

Supplement: Supplementary Figure 3 — Cladogram indicating the phylogenetic distribution of differentially abundant bacterial lineages in fecal samples between obese individuals before and after bilio-intestinal bypass, determined by linear discriminant analysis (LDA). Red dots represent bacterial groups over-abundant in post-surgery samples, while green dots represent bacterial groups overrepresented in pre-surgery samples. [file Image3.TIF]

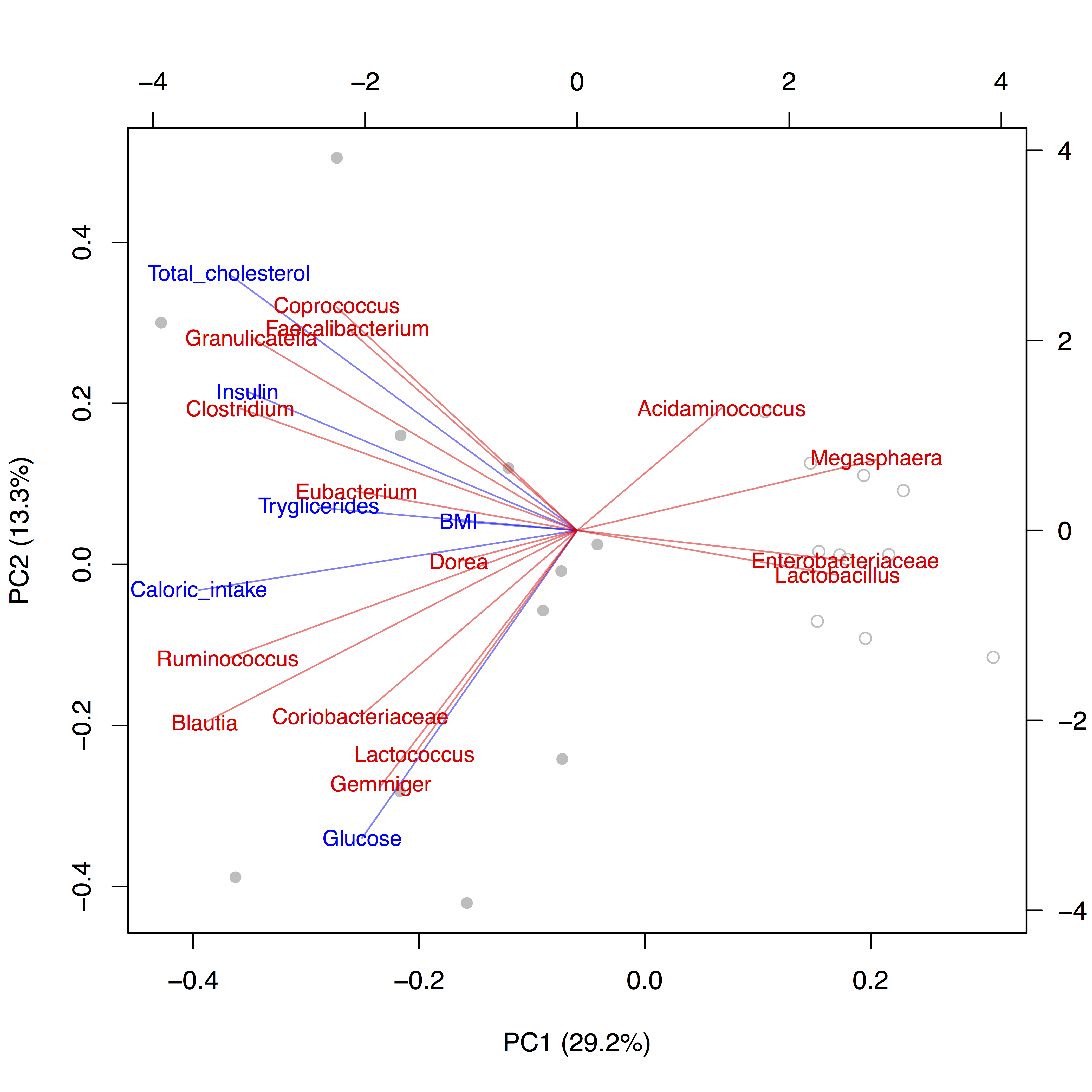

Supplement: Supplementary Figure 4 — Biplot of the first two principal components from the principal component analysis (PCA) of bacterial genera relative abundances and bio-clinical markers. The length of the vectors represents the PCA loadings of the variables on the first two principal components. Dots represent individual subjects before (solid circles) and after (open circles) bilio-intestinal bypass. [file Image4.TIFF]
